# Doctors Aboard Ship

**Published:** 1906-09-22

**Authors:** 


### DOCTORS ABOARD SHIP.

In your annotations in your paper last week it is stated that passengers sometimes refrain from utilising the services of the ship's doctor on account of the expected fee. Although I have never met with such a circumstance, I am aware that passengers have cause for complaint. In the majority of cases inefficient medical attention is all that they can rely on, however serious may be the necessity. During two out of three recent voyages I have had an opportunity of witnessing this. In one instance the doctor had had no other practical experience than that afforded by ten years' service on board ship, and he had evidently studied no medical literature of a later date. His advice for influenza was bleeding, for sea-sickness a glass of sea-water every morning. I have seen him, though desiring his patient to take solid food, insisting upon her consuming a cup of hot milk followed by a hot soup immediately before a meal. In fact, his advice not only was opposed to all accepted ideas in medicine, but was equally opposed to common sense. In the other instance the doctor's conduct was characterised by neglect and an absence of a sense of responsibility. He allowed a family suffering from ophthalmia to mix amongst the many children on the ship, and only segregated them when threatened with exposure in the Press by a lady fearful for her only child. These two doctors were not young men. The newly qualified man fresh from the schools I encountered on the third voyage was by far the most efficient. By this last remark I am by no means advocating the inexperienced medical man as a class to occupy the post of doctor on a ship absent for a long voyage, and containing a large number of passengers who may suffer from all manner of complaints. No doubt it is a difficult matter to secure the efficient and experienced, but until such can be obtained the passenger will suffer.

Voyager.

